# Supplementary material for: HIF-1α and HIF-2α differently regulate tumour development and inflammation of clear cell renal cell carcinoma in mice
Source: Nat Commun. 2020 Aug 17;11:4111. doi: 10.1038/s41467-020-17873-3 (PMC7431415; doi:10.1038/s41467-020-17873-3)
Supplement: Supplementary file 1 — Supplementary Information [file 41467_2020_17873_MOESM1_ESM.pdf]

Supplementary Information

Hoefflin *et al.*, HIF-1α and HIF-2α differently regulate tumour development, metabolism and inflammation of ccRCC in mice

Supplementary Figures 1-14

Supplementary Table 1

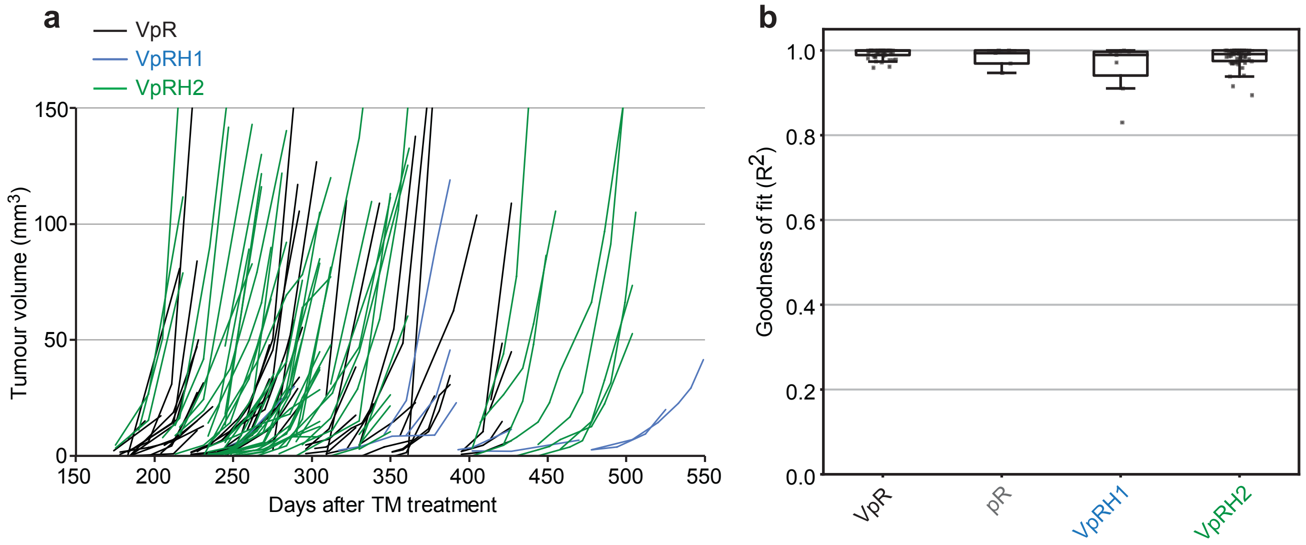

Supplementary Figure 1. Validation of the exponential nature of tumour growth curves. **a** Growth curves for VpR, VpRH1 and VpRH2 tumours after tamoxifen (TM) feeding. **b** Coefficients of determination (R^2) for exponential linear regression of single tumor growth curves. Values close to 1 show that the tumour growth data is well approximated by an exponential model (VpR n=48, pR n=5, VpRH1 n=7 and VpRH2 n=56). Box-whisker plots depict median, bounded by Q1 (25% lower quartile) and Q3 (75% upper quartile) and whiskers depict 1.5 times the Q3-Q1 interquartile range.

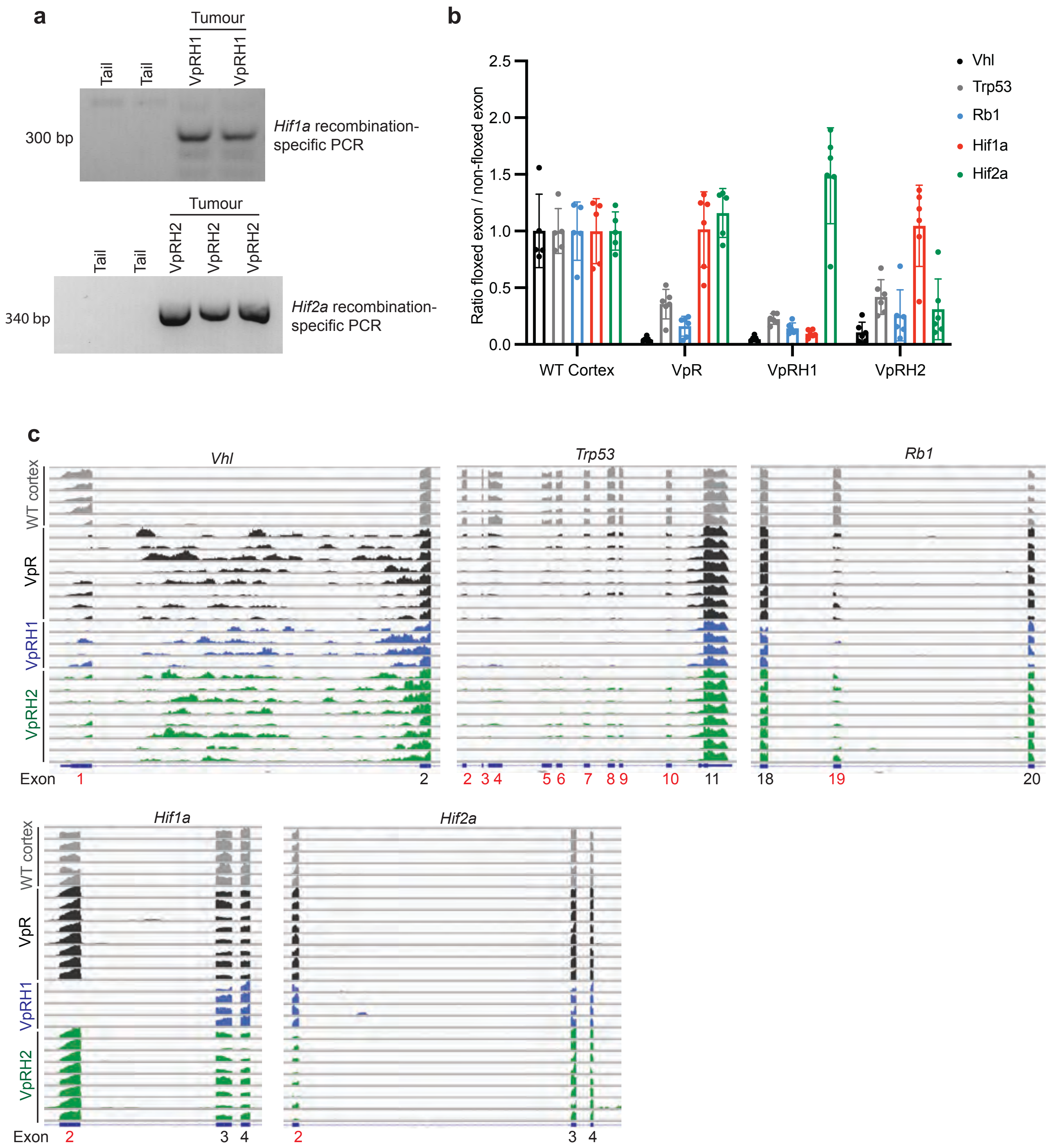

Supplementary Figure 2. Validation of Cre-mediated recombination of floxed genes in tumours. **a** PCRs from genomic DNA isolated from tails, VpRH1 (n=2) or VpRH2 tumours (n=3). The PCRs specifically amplify DNA fragments from the *Hif1a* and *Hif2a* loci only following Cre-mediated recombination of *lox*P sites. **b** Real time PCR-based analysis of the allelic frequencies of the floxed exons of *Vhl*, *Trp53*, *Rb1*, *Hif1a* and *Hif2a* genes (normalised to non-floxed exons which serve as DNA loading controls) using genomic DNA isolated from WT Cortex (n=5), VpR (n=6), VpRH1 (n=6) or VpRH2 (n=6) tumours. Mean ± std. dev. is shown. **c** Representative RNA sequencing traces for the *Vhl*, *Trp53*, *Rb1*, *Hif1a* and *Hif2a* genes in RNA isolated from WT Cortex, VpR, VpRH1 or VpRH2 tumours. Floxed exons are labelled in red, non-floxed exons in black. Y-axes are internally normalised for each track to facilitate comparisons of the relative sequencing coverage of adjacent exons in each sample.

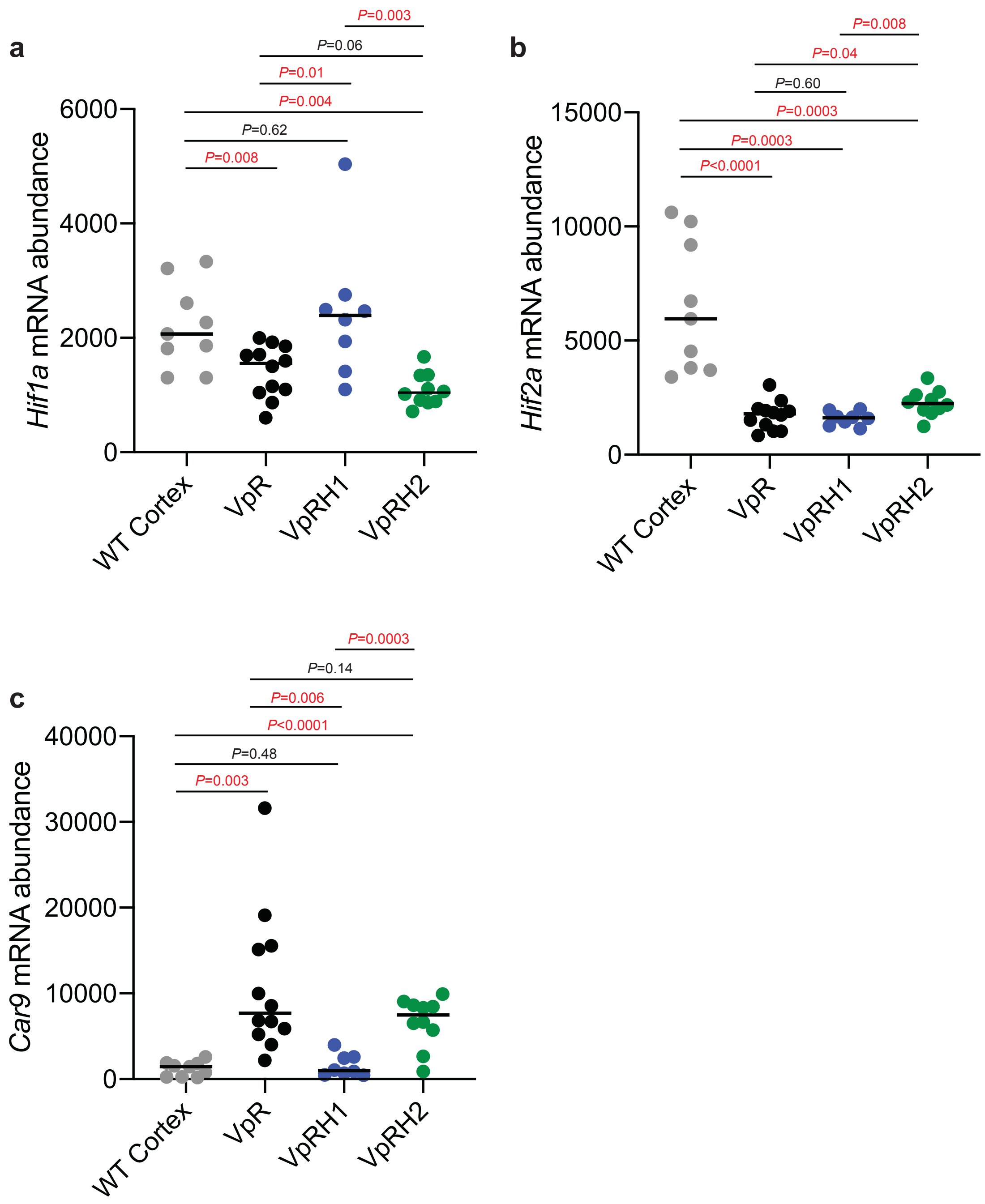

Supplementary Figure 3. Analyses of *HIf1a*, *Hif2a* and *Car9* mRNA expression. **a-c** Distributions and mean expression levels of *Hif1a*, *Hif2a* and *Car9* in WT Cortex (n=9), VpR (n=12), VpRH1 (n=8) and VpRH2 (n=10) tumours derived from RNA sequencing analyses. Two-sided *P* values were calculated by Student's t-test.

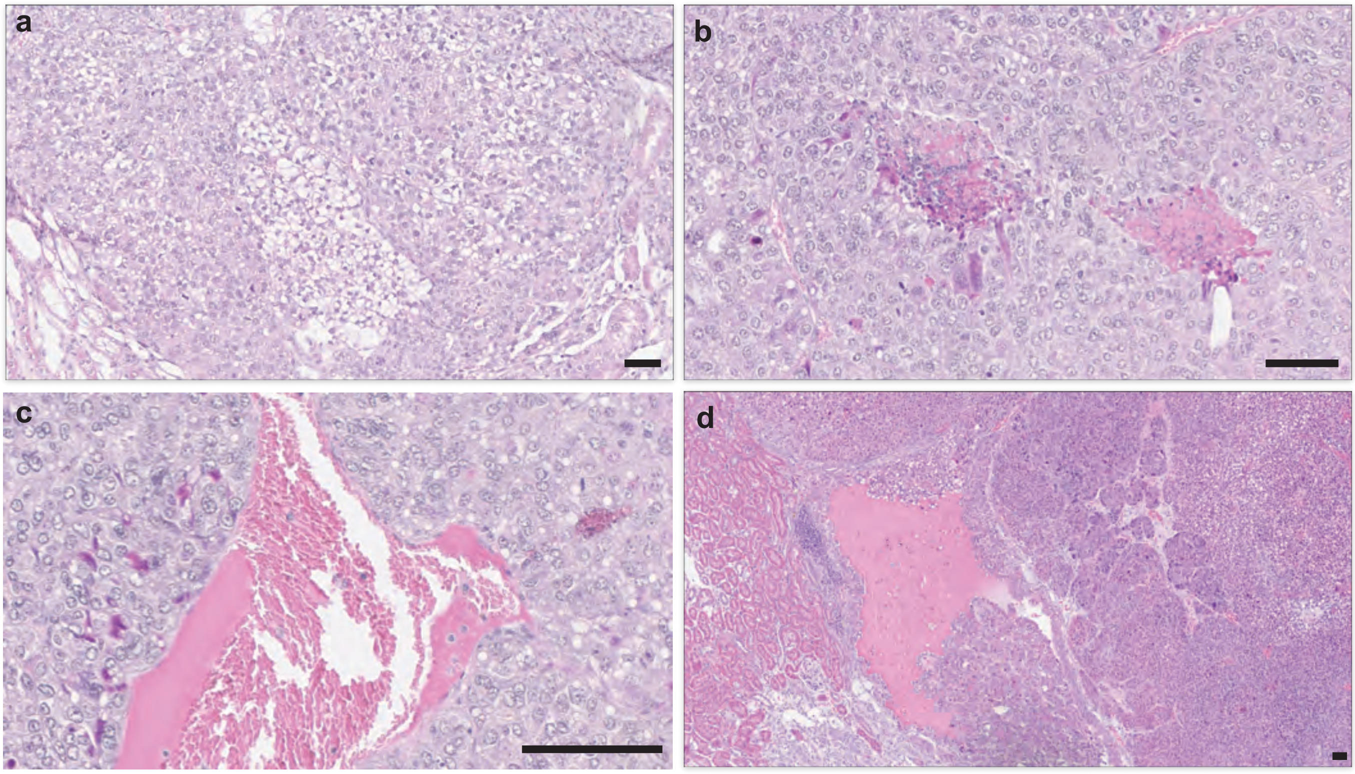

Supplementary Figure 4. Examples of tumour phenotypes. **a** Histomorphological heterogeneity in a VpR tumour. This image is representative of 23% of VpR and 28% of VpRH2 tumours. **b** Necrosis in a VpR tumour. **c** Hemorrhage in a VpR tumour. Images in **b** and **c** are representative of similar regions of necrosis and hemorrhage that were observed in many larger VpR, VpRH1 and VpRH2 tumours. **d** Tumour with cyst in a VpRH2 mouse. Similar cysts were observed in 15% of VpR tumours, 25% of VpRH1 tumours and 19% of VpRH2 tumours. Scale bars depict 100 μm. 26 VpR, 16 VpRH1 and 21 VpRH2 tumours were analysed.

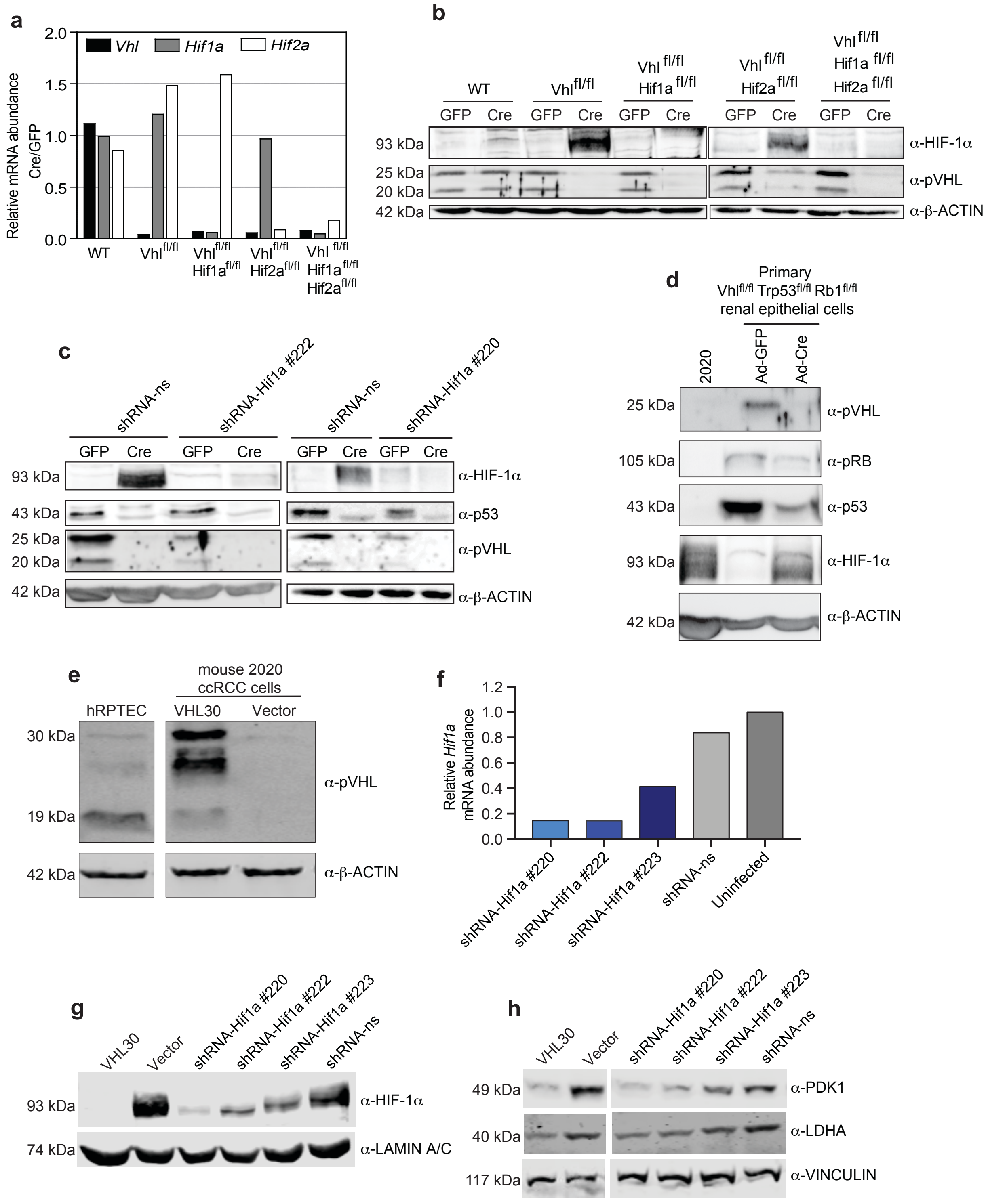

Supplementary Figure 5. Validation of genetic manipulations in MEFs and 2020 mouse ccRCC cells. **a** Real time PCR analysis of mRNA abundance of *Vhl*, *Hif1a* and *Hif2a* in MEFs of the indicated floxed genotypes. Data represent the ratio of Adeno-Cre infected cells to Adeno-GFP infected cells, normalised to 12S ribosomal RNA. **b** Western blotting of the cells from A for HIF-1α, pVHL and β-ACTIN (loading control). **c** Western blotting of *Vhl$^{fl/fl}$Trp53$^{fl/fl}$* MEFs for HIF-1α, p53, pVHL and β-ACTIN after infection with lentiviral shRNA-ns, shRNA-*Hif1a* #220 or shRNA-*Hif1a* #222 followed by infection with Adeno-Cre or Adeno-GFP. **d** Western blotting of mouse ccRCC cell line 2020 for HIF-1α, p53, pRB, pVHL and β-ACTIN. Primary renal epithelial cells derived from *Vhl$^{fl/fl}$Trp53$^{fl/fl}$Rb1$^{fl/fl}$* mice infected with Adeno-Cre or Adeno-GFP served as controls. **e** Western blotting for pVHL and β-ACTIN in mouse ccRCC cell line 2020 infected with empty vector or re-expressing human VHL30. Human renal proximal tubule epithelial cells (RPTEC) served as a positive control for pVHL19 and pVHL30 expression. **f** Real time PCR analysis of mRNA abundance of *Hif1a* in mouse ccRCC 2020 cells infected with shRNA-ns, shRNA-*Hif1a* #220, shRNA-*Hif1a* #222 or shRNA-*Hif1a* #223. Data were normalised to 12S ribosomal RNA levels. **g** Western blotting for HIF-1α and LAMIN A/C (loading control) in cells described in e and f. **h** Western blotting for PDK1, LDH-A and VINCULIN (loading control) in cells described in e and f. Molecular analyses in a-h were conducted once on the starting cell populations of each experiment, which were subsequently analysed in the replicate experiments shown in Fig. 2.

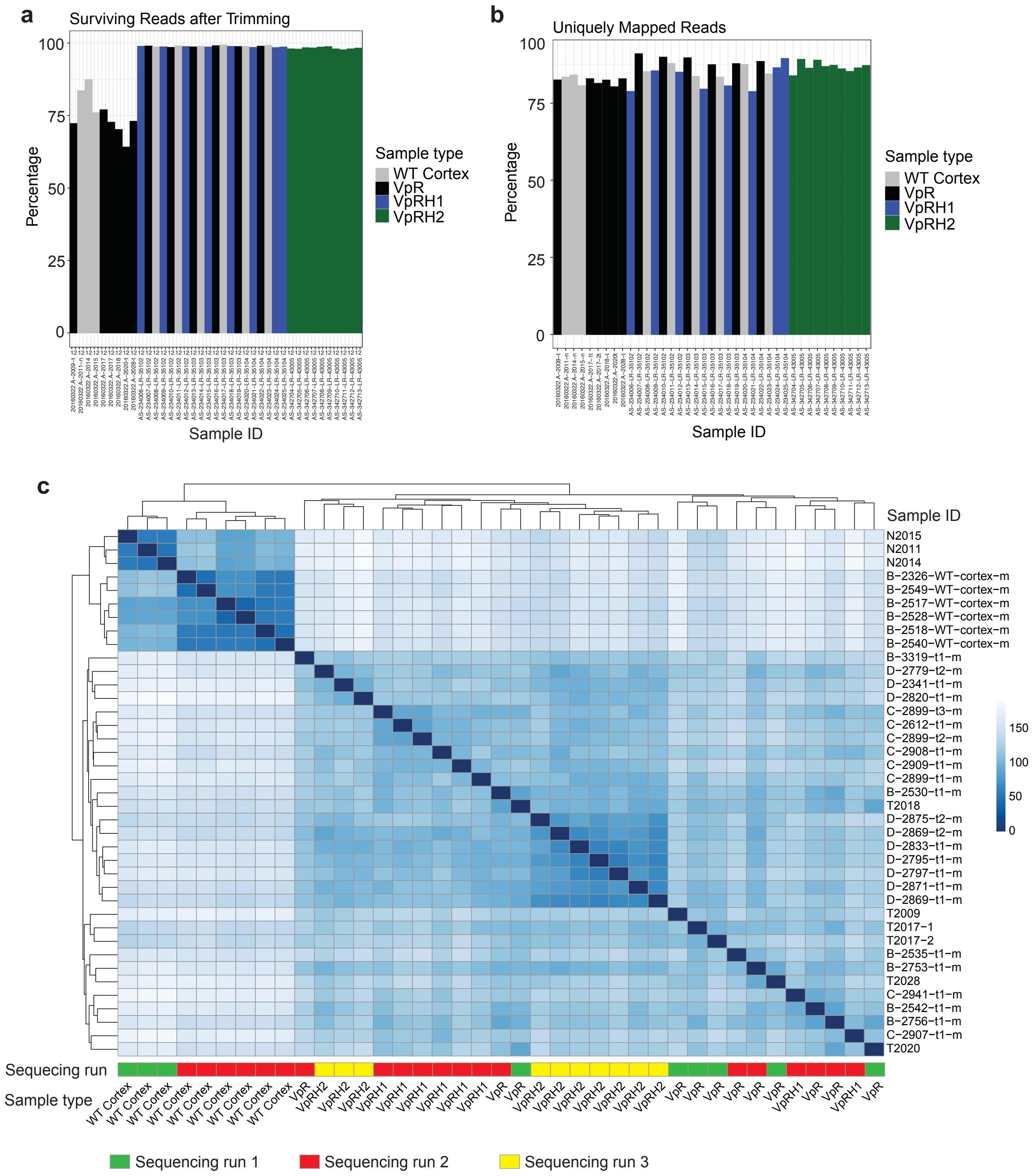

Supplementary Figure 6. Quality control of RNA sequencing data. **a** Read trimming. **b** Read alignment. **c** Unsupervised hierarchical clustering by sample Euclidean distance matrix analysis.

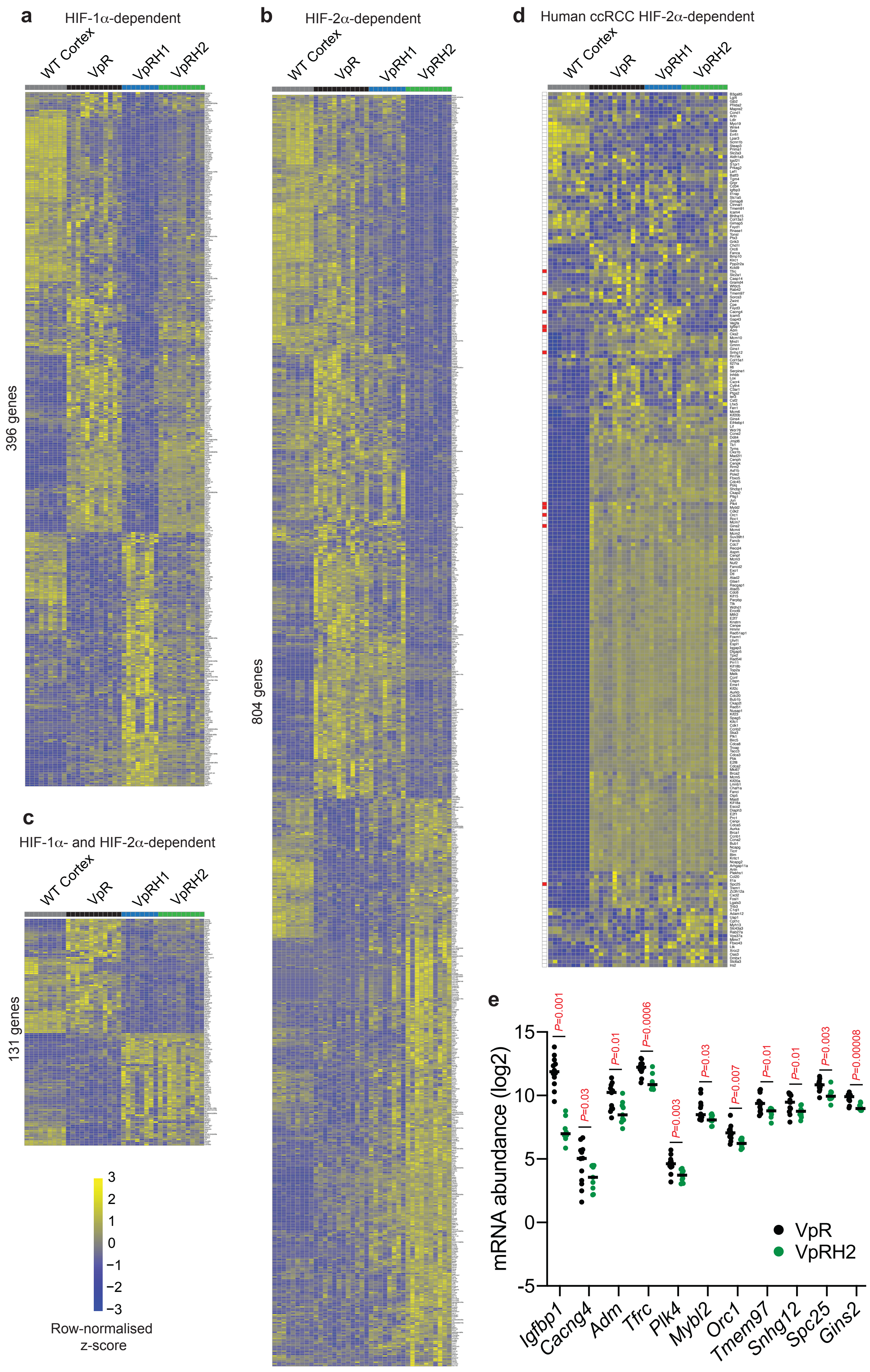

a
HIF-1α-dependent
WT Cortex
VpR
VpRH1
VpRH2
396 genes
b
HIF-2α-dependent
WT Cortex
VpR
VpRH1
VpRH2
804 genes
c
HIF-1α- and HIF-2α-dependent
WT Cortex
VpR
VpRH1
VpRH2
131 genes
d
Human ccRCC HIF-2α-dependent
WT Cortex
VpR
VpRH1
VpRH2
3
2
1
0
−1
−2
−3
Row-normalised
z-score
e
mRNA abundance (log2)
15
10
5
0
-5
P=0.001
P=0.03
P=0.01
P=0.0006
P=0.003
P=0.03
P=0.007
P=0.01
P=0.01
P=0.003
P=0.00008
Igfbp1
Cacng4
Adm
Tfrc
Plk4
Mybl2
Orc1
Tmem97
Snhg12
Spc25
Gins2
VpR
VpRH2

Supplementary Figure 7. Analyses of RNA sequencing. **a** Heatmaps of mRNA abundance for differentially expressed genes between VpRH1and VpR tumours. **b** Heatmap of mRNA abundance for differentially expressed genes between VpRH2 and VpR tumours. **c** Heatmap of mRNA abundance for genes that are differentially regulated in both VpRH1 and VpRH2 compared to VpR tumours. **d** Heatmap of mRNA abundance of mouse orthologues of human ccRCC HIF-2α-dependent genes. Red squares highlight genes that are HIF-2α-dependent in the mouse dataset. Rows represent row-normalised z-scores of mRNA abundance, each column represents an individual sample from WT cortex or VpR, VpRH1 and VpRH2 tumours. All heatmaps have the same z-score scale. **e** Distributions and mean expression levels of the red-marked genes from **d** in VpR (n=12) and VpRH2 (n=10) tumours derived from RNA sequencing analyses. Two-sided *P* values were calculated by Student's t-test.

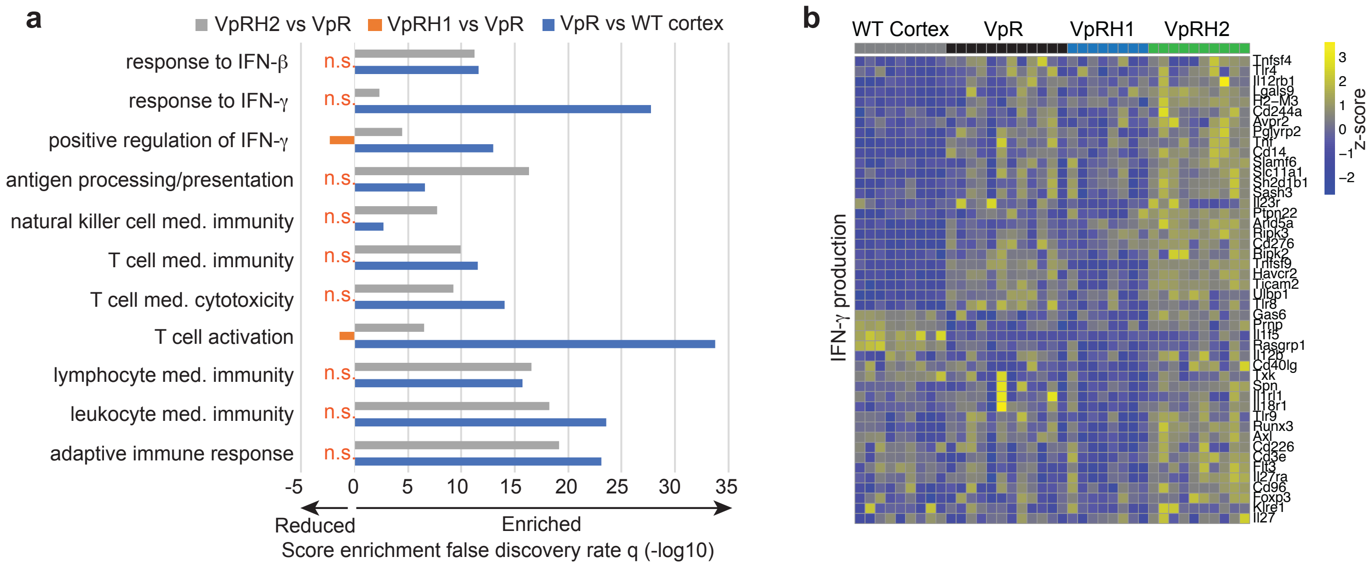

Supplementary Figure 8. Immune infiltration and Interferon-γ pathways are upregulated in VpRH2 tumours. **a** Pairwise GAGE comparisons for the indicated gene sets of VpR versus WT cortex, VpRH1 versus VpR and VpRH2 versus VpR tumours. The false discovery rate for each of the differentially expressed GAGE signatures is shown. n.s. represents no significant difference in the comparison. **b** Gene expression heatmap for the GAGE term IFN-γ production. Rows represent row-normalised z-scores of mRNA abundance, each column represents an individual sample from WT cortex or VpR, VpRH1 and VpRH2 tumours.

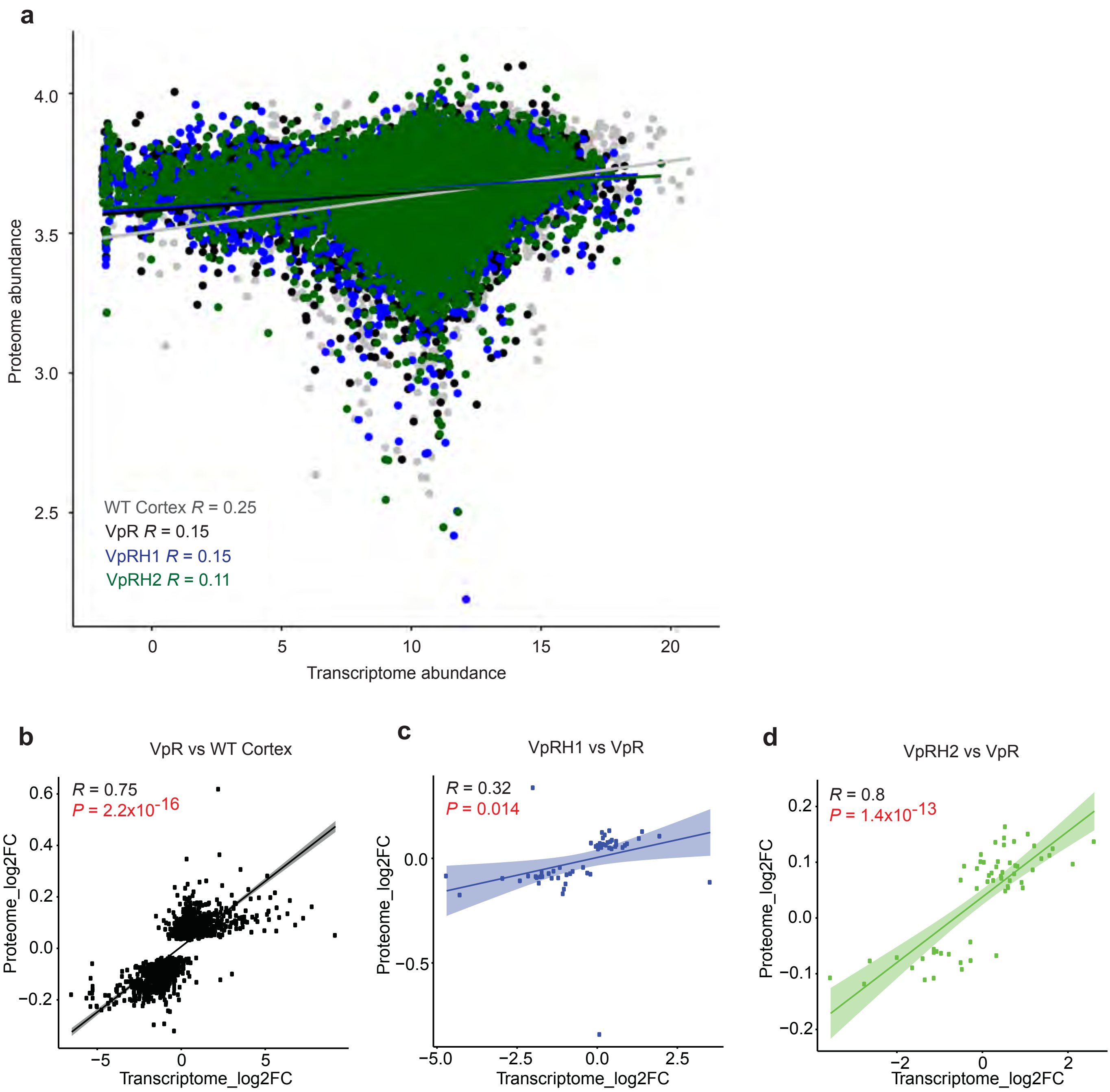

Supplementary Figure 9. Correlation analyses of protemic and transcriptomic data. **a** Dots represent the absolute protein and mRNA expression levels of individual genes, coloured by sample type. **b-d** Dots represent the fold changes in protein and mRNA abundance of individual genes in the comparison WT Cortex vs VpR (**b**), VpRH1 vs VpR (**c**) and VpRH2 vs VpR (**d**). Only genes for which the protein showed a statistically significant ($P<0.01$) difference in expression in the comparison were included in the analysis. Shaded areas represent the 95% confidence interval of the linear regression. For VpR vs WT Cortex 1418 gene/protein pairs were analysed, for VpRH1 vs VpR 59 gene/protein pairs were analysed and for VpRH2 vs VpR 57 gene/protein pairs were analysed. Pearson correlation statistics and significances are shown.

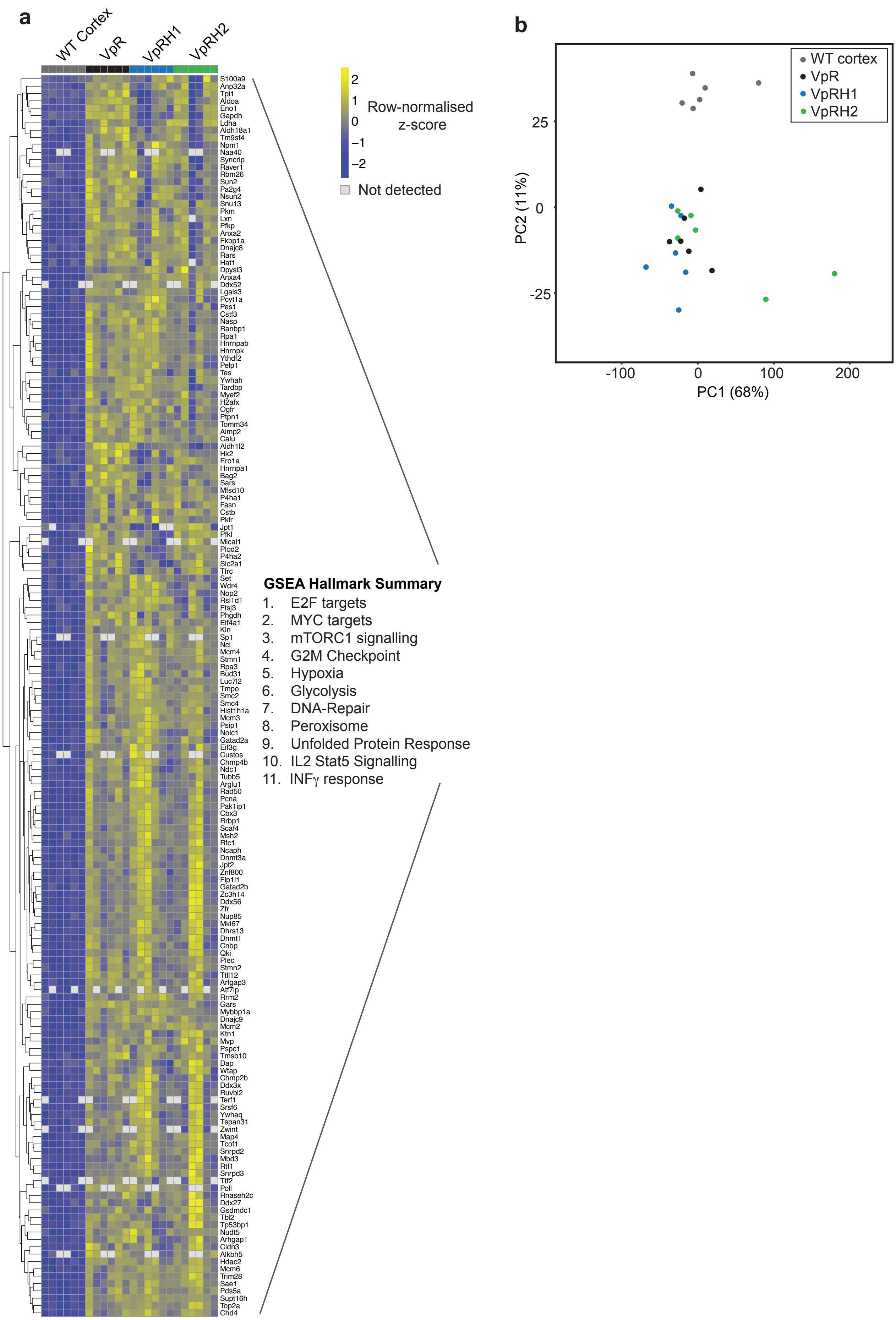

Supplementary Figure 10. Analyses of proteomics. **a** Protein expression heatmap for proteins that are differentially overexpressed in VpR tumours compared to WT Cortex, together with a summary of GSEA Hallmark terms associated with these proteins. **b** Principal component analysis of proteomics of WT Cortex and VpR, VpRH1 and VpRH2 tumours.

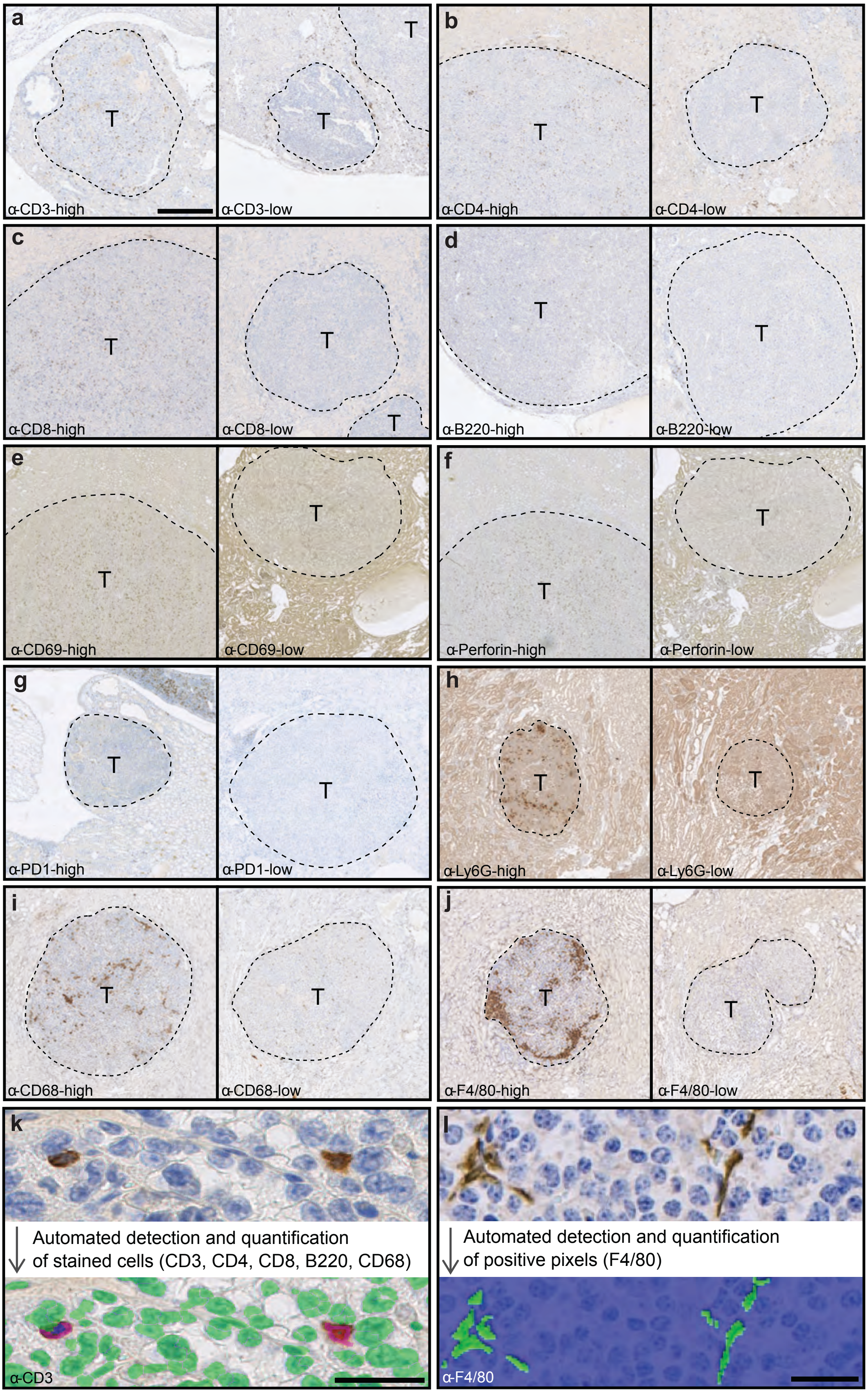

Supplementary Figure 11. Analysis of immune cell infiltration in tumours. **a-j** Examples of immunohistochemical stainings in tumours showing high and low densities of cells for each of the indicated marker proteins. The border between tumour (T) and normal is highlighted by a dotted line. All images are the same magnification, scale bar = 500 μm **k** Example of the automated detection of nuclei (green) and stained cells (purple). **l** Example of automated positive pixel detection of stained cells (green). Scale bars in k,l = 50 μm. Images in a-l are representative of stainings that were used for the quantifications depicted in Fig. 6b-k.

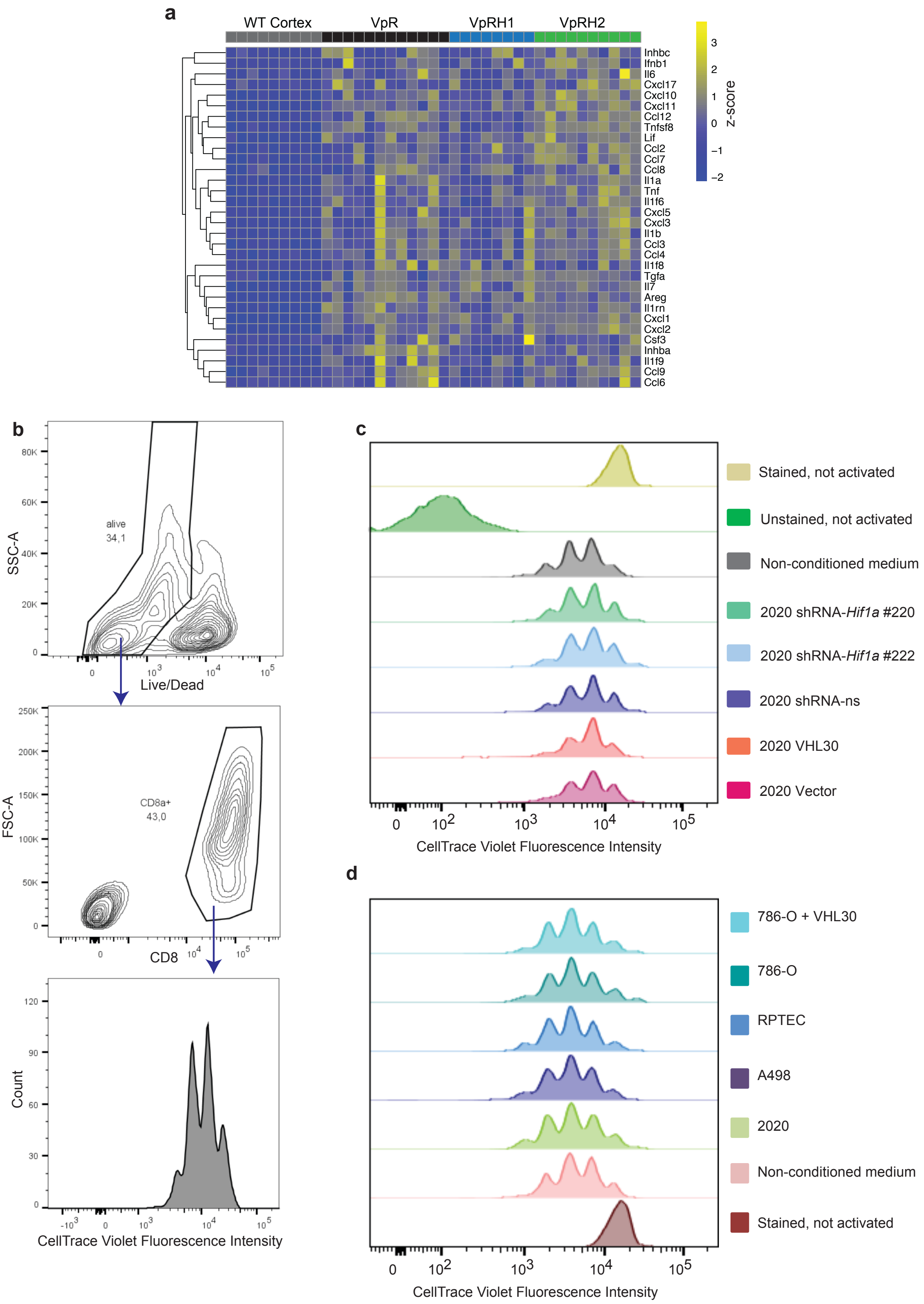

Supplementary Figure 12. Investigation of potential mechanisms of crosstalk between ccRCC cells and CD8^{+} T cells**. a** Heatmap of mRNA abundance for differentially expressed cytokine genes. **b** Flow cytometry gating strategy for analyses in c and d. **c,d** Conditioned medium was generated by culturing the indicated mouse and ccRCC cell lines for 48 hours prior to addition of purified mouse splenic CD8^{+} T cells that were pre-activated with CD3/CD28 beads plus IL-2 and labelled with CellTrace Violet. Cells were harvested after 3 days for flow cytometry analyses, gated on CD8^{+} cells, where T cell proliferation is indicated by the shift in peaks from right to left due to the dilution of the dye. **c** Conditioned medium was made from the indicated genetically modified mouse ccRCC 2020 cells and compared to non-conditioned medium. Stained and unstained but not activated T cells served as controls. **d** Conditioned medium was made from mouse ccRCC 2020 cells, human renal proximal tubule epithelial cells (RPTEC), human ccRCC cell lines A498, 786-O as well as 786-O cells re-expressing VHL30 and compared to non-conditioned medium. Stained but not activated T cells served as controls.

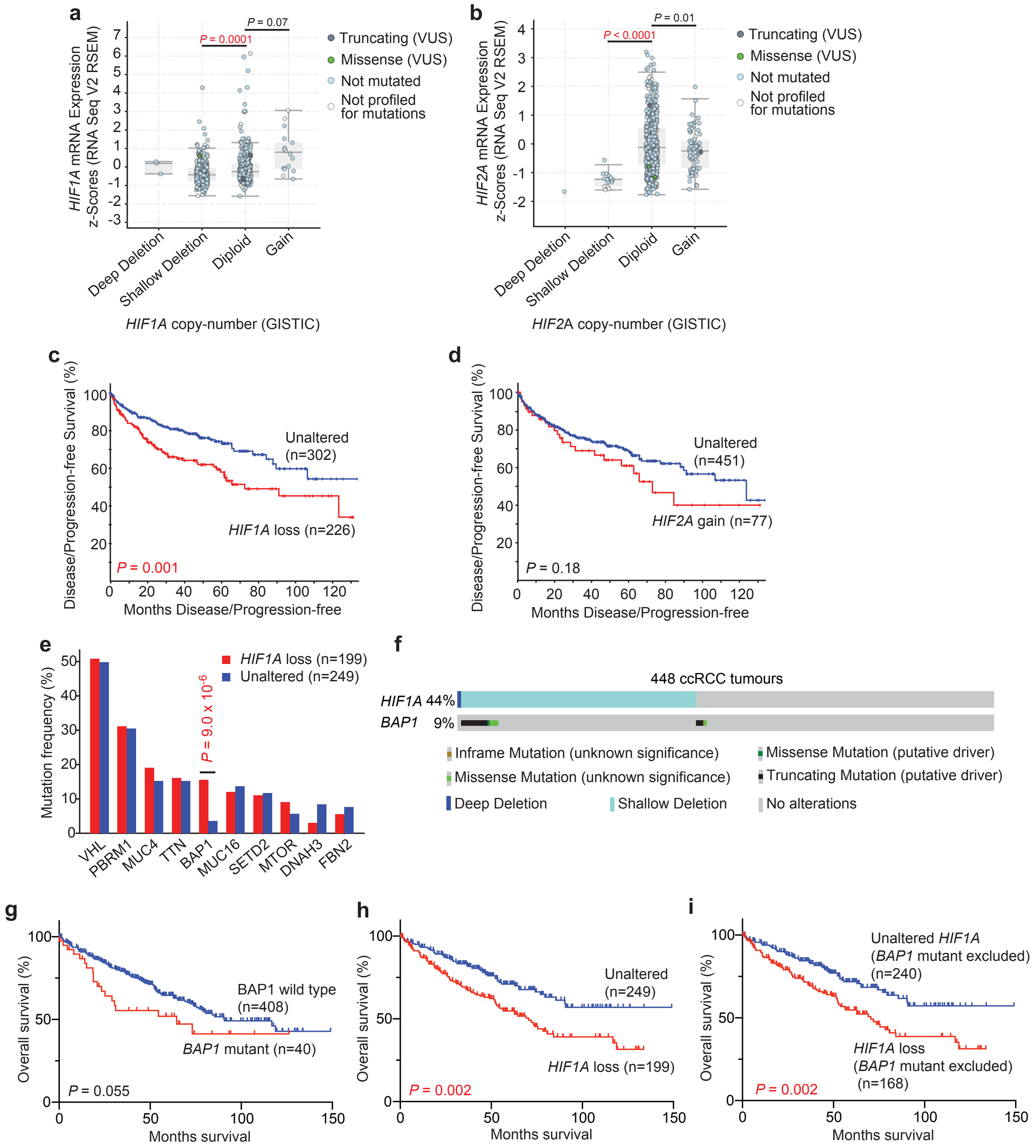

Supplementary Figure 13. Additional analyses of human ccRCC (TCGA KIRC dataset) related to Figure 7. **a,b** mRNA abundance of *HIF1A* (**a**) and *HIF2A* (**b**) in relation to copy number status. Box-whisker plots depict median, bounded by Q1 (25% lower quartile) and Q3 (75% upper quartile) and whiskers depict 1.5 times the Q3-Q1 interquartile range. *P* values were calculated by two-sided Student's t-test. **c,d** Kaplan-Meier curves showing disease/progression-free survival of ccRCC patients whose tumours exhibit loss of one or two copies of *HIF1A* (loss) (**c**) or gain of a copy of *HIF2A* (gain) (**d**) versus patients without these copy number alterations (Unaltered). *P* values are derived from the two-sided log-rank test. **e** Distributions of the frequencies of mutation of the most commonly mutated genes in ccRCC in the *HIF1A* loss and unaltered subclassifications. *P* value is from Fischer's exact test. **f** Oncoprint showing the distribution of copy losses of *HIF1A* and mutations of *BAP1*. **g-i** Kaplan-Meier curves showing overall survival of ccRCC patients based on *HIF1A* copy number status (**g**), *BAP1* mutation status (**h**) or *HIF1A* copy number status in the dataset where all *BAP1* mutant tumours have been excluded (**i**). *P* values are derived from the two-sided log-rank test.

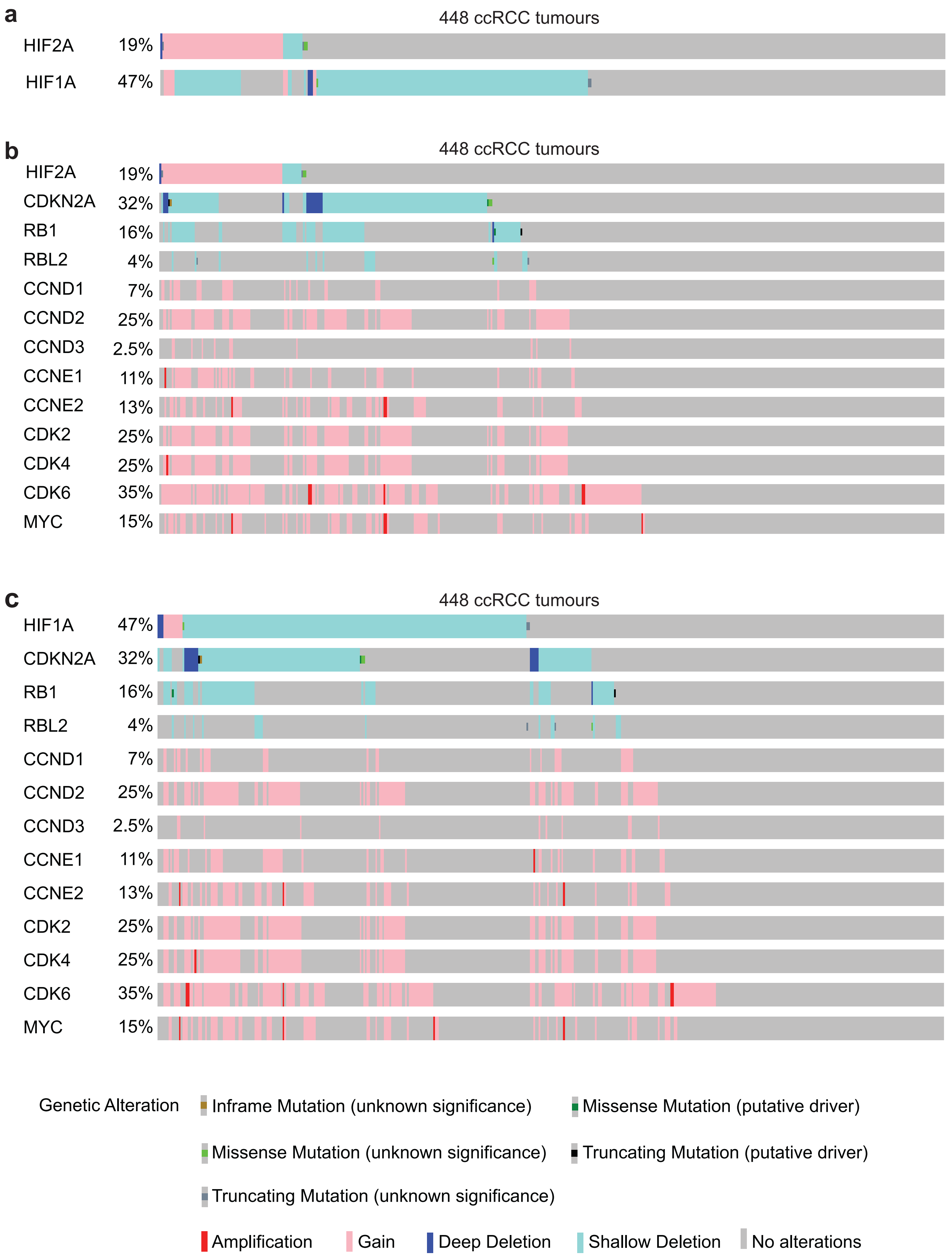

Supplementary Figure 14. Correlation of copy number changes in HIF genes and cell cycle regulatory genes **a-c** Oncoprints of genetic alterations in the indicated genes in the TCGA (KIRC) ccRCC data set of 448 tumours.

Supplementary Table 1: Statistically significant co-variants of *HIF1A* loss in ccRCC

**Clinical Attribute**	**Attribute Type**	**Statistical Test**	**p-Value**	**q-Value**
Sex	Patient	Chi-squared Test	0	0
Fraction Genome Altered	Sample	Kruskal Wallis Test	3.98E-11	1.23E-09
Neoplasm Histologic Grade	Patient	Chi-squared Test	1.69E-07	3.50E-06
Neoplasm Disease Stage American Joint Committee on Cancer Code	Patient	Chi-squared Test	7.46E-06	1.16E-04
Patient's Vital Status	Patient	Chi-squared Test	1.28E-05	1.59E-04
Overall Survival Status	Patient	Chi-squared Test	2.42E-05	2.51E-04
Disease Free Status	Patient	Chi-squared Test	3.23E-05	2.86E-04
Neoplasm Disease Lymph Node Stage American Joint Committee on Cancer Code	Patient	Chi-squared Test	6.07E-04	4.70E-03
American Joint Committee on Cancer Tumor Stage Code	Patient	Chi-squared Test	9.11E-04	6.28E-03
American Joint Committee on Cancer Metastasis Stage Code	Patient	Chi-squared Test	2.92E-03	0.0181
Lymph nodes examined positive	Patient	Chi-squared Test	5.67E-03	0.0319
