# Supplementary material for: HIF-1α and HIF-2α differently regulate tumour development and inflammation of clear cell renal cell carcinoma in mice
Source: Nat Commun. 2020 Aug 17;11:4111. doi: 10.1038/s41467-020-17873-3 (PMC7431415; doi:10.1038/s41467-020-17873-3)
Supplement: Supplementary file 3 — Descriptions of Additional Supplementary Files [file 41467_2020_17873_MOESM3_ESM.pdf]

Description of additional supplementary files

Supplementary Data 1

Description: RNA sequencing

Supplementary Data 2

Description: GAGE analyses

Supplementary Data 3

Description: Proteomic analyses

Supplementary Data 4

Description: Correlation analyses of expression of HIF2A and antigen presentation machinery

Supplementary Data 5

Description: Summary of gene signatures used for ssGSEA immune cell deconvolution Seite 5/6

Supplementary Data 6

Description: Immune cell deconvolution z-scores and P-values for Figs. 6a and 7n
