# Supplementary material for: HIF-1α and HIF-2α differently regulate tumour development and inflammation of clear cell renal cell carcinoma in mice
Source: Nat Commun. 2020 Aug 17;11:4111. doi: 10.1038/s41467-020-17873-3 (PMC7431415; doi:10.1038/s41467-020-17873-3)
Supplement: Supplementary file 10 — Reporting Summary [file 41467_2020_17873_MOESM10_ESM.pdf]

nature research

Corresponding author(s): Frew

Last updated by author(s): Jul 9, 2020

Reporting Summary

Nature Research wishes to improve the reproducibility of the work that we publish. This form provides structure for consistency and transparency in reporting. For further information on Nature Research policies, see Authors & Referees and the Editorial Policy Checklist .

Statistics

For all statistical analyses, confirm that the following items are present in the figure legend, table legend, main text, or Methods section.

n/a	Confirmed	
☐	☒	The exact sample size (n) for each experimental group/condition, given as a discrete number and unit of measurement
☐	☒	A statement on whether measurements were taken from distinct samples or whether the same sample was measured repeatedly
☐	☒	The statistical test(s) used AND whether they are one- or two-sided<br>*Only common tests should be described solely by name; describe more complex techniques in the Methods section.*
☒	☐	A description of all covariates tested
☒	☐	A description of any assumptions or corrections, such as tests of normality and adjustment for multiple comparisons
☐	☒	A full description of the statistical parameters including central tendency (e.g. means) or other basic estimates (e.g. regression coefficient) AND variation (e.g. standard deviation) or associated estimates of uncertainty (e.g. confidence intervals)
☐	☒	For null hypothesis testing, the test statistic (e.g. F, t, r) with confidence intervals, effect sizes, degrees of freedom and P value noted<br>*Give P values as exact values whenever suitable.*
☒	☐	For Bayesian analysis, information on the choice of priors and Markov chain Monte Carlo settings
☐	☒	For hierarchical and complex designs, identification of the appropriate level for tests and full reporting of outcomes
☐	☒	Estimates of effect sizes (e.g. Cohen's d, Pearson's r), indicating how they were calculated

Our web collection on statistics for biologists contains articles on many of the points above.

Software and code

Policy information about availability of computer code

Data collection

Analyses on human ccRCC Data were performed using the TCGA KIRC Firehose-legacy data-set [http://firebrowse.org/?cohort=KIRC&download_dialog=true] using R.Studio (v.4.0.2) and the respective packages (see Data analysis) and cBioPortal for Cancer Genomics [https://www.cbioportal.org].

Data analysis

RNA-Sequencing:
Raw data fastq-files were pre-processed with trimmomatic (v0.39) to assure sufficient read quality by removing adapters and bases in the low quality segment regions (end of the reads) with a base quality below 20. After quality control and trimming the reads were 2-pass aligned using the STAR aligner (v2.7.0a) and the GRCm38 reference genome from Ensembl. The alignment step was followed by normalization and differential expression analysis with the R/Bioconductor package (v.3.11) DESeq2 (v.1.28.1) After pre-processing and filtering 19,723 genes were further analysed and fitted with a negative binomial generalised linear model followed by Wald statistics to identify differentially expressed genes. Genes were considered significant with an adjusted p-value < 0.001 (Benjamini-Hochberg).
Gene Set Enrichment Analysis:
Enrichment of signalling pathways was performed as implemented in the R/Bioconductor package GAGE (Generally Applicable Gene-Set Analysis, v2.37.0) with signalling pathways from Gene Ontology, ConsensusPathDB, and MSigDB. The human gene identifiers from the MSigDB pathways were mapped on mouse homologs with the R/Bioconductor package GeneAnswers (v2.28.0). Pathways were considered significant with an adjusted p-value < 0.05 (Benjamini-Hochberg).
ssGSEA Immune Deconvolution Analysis:
RNA-seq raw read sequences were aligned against mouse genome assembly mm10 by STAR 2-pass alignment (v2.7.0a) QC metrics, for example general sequencing statistics, gene feature and body coverage, were then calculated based on the alignment result through RSeQC (v2.6.4). RNA-seq gene level count values were computed by using the R package GenomicAlignments (1.24.0) over aligned reads with UCSC KnownGene in mm10 as the base gene model. The Union counting mode was used and only mapped paired reads after alignment quality filtering were considered. Gene level FPKM (Fragments Per Kilobase Million) and raw read count values were computed by the R package DESeq2 (v.1.28.1) Single-Sample GSEA was utilised for immune deconvolution analyses based on FPKM expression values to estimate the abundance of immune cell types, MHC class I antigen presenting machinery expression, T-cell infiltration score (TIS), Immune Infiltration Score (IIS) and immune cytolytic score (CYT) as well as the eTME signatures which was developed from

leveraging RCC patient-derived xenograft RNA-sequencing data. In addition to the gene signature-based deconvolution approach, CIBERSORT which is a regression-based method using Support Vector Machine algorithm was also employed using either the human gene panel or the mouse specific reference panel, ImmuCC.
Proteomics:
Data were analyzed by MaxQuant (v1.6.013) with the following settings: tryptic specificity, up to two missed cleavages, TMT-modification of peptide N-termini and lysine side chains; cysteine carbamidomethylation, mouse reviewed sequences (downloaded from Uniprot on Aug 26th, 2019), 1 % FDR for peptides and proteins, precursor intensity fraction = 0.5, one or more unique peptides for protein quantitation. MaxQuant output was further processed by MSStatsTMT (v1.6.3) for normalisation, batch removal, and protein assembly. Differential protein abundance was assessed using linear models of microarray analysis.
Image Quantification:
For analyses of immune cell markers sections were scanned using a Nanozoomer Scansystem (Hamamatsu Photonics). Automatic quantifications was performed using the VIS software suite (Visiopharm, Hoersholm, Denmark, v4.6.1.630).

For manuscripts utilizing custom algorithms or software that are central to the research but not yet described in published literature, software must be made available to editors/reviewers. We strongly encourage code deposition in a community repository (e.g. GitHub). See the Nature Research guidelines for submitting code & software for further information.

Data

Policy information about availability of data

All manuscripts must include a data availability statement. This statement should provide the following information, where applicable:

- Accession codes, unique identifiers, or web links for publicly available datasets
- A list of figures that have associated raw data
- A description of any restrictions on data availability

Raw proteomics data are available via PRIDE / ProteomeXchange with identifier PXD016630 [http://www.ebi.ac.uk/pride/archive/projects/PXD016630]. Raw RNA sequencing data have been uploaded to GEO with identifier GSE150983 [https://www.ncbi.nlm.nih.gov/geo/query/acc.cgi?acc=GSE150983]. Analyses on human ccRCC Data were performed using the TCGA KIRC Firehose-legacy data-set [http://firebrowse.org/?cohort=KIRC&download_dialog=true]. Source data are provided with this paper. All remaining relevant data are available in the article, supplementary information or from the corresponding author upon reasonable request.

Field-specific reporting

Please select the one below that is the best fit for your research. If you are not sure, read the appropriate sections before making your selection.

☒ Life sciences ☐ Behavioural & social sciences ☐ Ecological, evolutionary & environmental sciences

For a reference copy of the document with all sections, see nature.com/documents/nr-reporting-summary-flat.pdf

Life sciences study design

All studies must disclose on these points even when the disclosure is negative.

Sample size	Statistical power tests (2 sided Log-Rank test, 80% power, 5% significance) were utilised to determine minimal sample sizes of mouse cohorts for Kaplan-Meier survival outcomes. Larger cohort sizes were in fact used.<br>Cellular assays comparing genotypes involved direct pairwise comparisons of isogenic sets of cells that had been genetically manipulated. Preceding sample-size calculations were not performed for these experiments due to their purely investigative nature where both the potential phenotypes and magnitude of effects could not be predicted in advance. Experiments for Fig 2a-b were performed in 3 independent cultures. Experiments for Fig. 2c-g in two independent experiments each with replicates of six cultures. In all of the cell culture experiments Mean and standard deviation were calculated and showed significant differences.
Data exclusions	No data were excluded from analyses.
Replication	Replication of cell culture experiments were successful and are described in the figure legends. All attempts at replication were successful.
Randomization	Randomisation was performed for mouse allograft experiments. Analyses of macroscopic and microscopic mouse phenotypes as well as RNA-Sequencing- and Proteomics-Analyses were dependent on the respective genetic background, therefore randomization was not possible or necessary.
Blinding	For practical reasons relating to the breeding, genotyping and generation of cohorts of tamoxifen-fed mutant mice, investigators were not blinded to the genotype for imaging experiments. However, for all other microscopic analyses, every mouse was identified solely using an individual Mouse-ID that did not provide information about the genetic background. The assignment to the respective genetic background was performed after scoring or quantification, hence these analyses were performed in a blinded manner. Cell culture experiments were not blinded, since the respective genetic manipulations had to be induced by the investigator and therefore could not be blinded.

Behavioural & social sciences study design

All studies must disclose on these points even when the disclosure is negative.

Study description	*Briefly describe the study type including whether data are quantitative, qualitative, or mixed-methods (e.g. qualitative cross-sectional, quantitative experimental, mixed-methods case study).*
Research sample	*State the research sample (e.g. Harvard university undergraduates, villagers in rural India) and provide relevant demographic information (e.g. age, sex) and indicate whether the sample is representative. Provide a rationale for the study sample chosen. For studies involving existing datasets, please describe the dataset and source.*
Sampling strategy	*Describe the sampling procedure (e.g. random, snowball, stratified, convenience). Describe the statistical methods that were used to predetermine sample size OR if no sample-size calculation was performed, describe how sample sizes were chosen and provide a rationale for why these sample sizes are sufficient. For qualitative data, please indicate whether data saturation was considered, and what criteria were used to decide that no further sampling was needed.*
Data collection	*Provide details about the data collection procedure, including the instruments or devices used to record the data (e.g. pen and paper, computer, eye tracker, video or audio equipment) whether anyone was present besides the participant(s) and the researcher, and whether the researcher was blind to experimental condition and/or the study hypothesis during data collection.*
Timing	*Indicate the start and stop dates of data collection. If there is a gap between collection periods, state the dates for each sample cohort.*
Data exclusions	*If no data were excluded from the analyses, state so OR if data were excluded, provide the exact number of exclusions and the rationale behind them, indicating whether exclusion criteria were pre-established.*
Non-participation	*State how many participants dropped out/declined participation and the reason(s) given OR provide response rate OR state that no participants dropped out/declined participation.*
Randomization	*If participants were not allocated into experimental groups, state so OR describe how participants were allocated to groups, and if allocation was not random, describe how covariates were controlled.*

Ecological, evolutionary & environmental sciences study design

All studies must disclose on these points even when the disclosure is negative.

Study description	*Briefly describe the study. For quantitative data include treatment factors and interactions, design structure (e.g. factorial, nested, hierarchical), nature and number of experimental units and replicates.*
Research sample	*Describe the research sample (e.g. a group of tagged Passer domesticus, all Stenocereus thurberi within Organ Pipe Cactus National Monument), and provide a rationale for the sample choice. When relevant, describe the organism taxa, source, sex, age range and any manipulations. State what population the sample is meant to represent when applicable. For studies involving existing datasets, describe the data and its source.*
Sampling strategy	*Note the sampling procedure. Describe the statistical methods that were used to predetermine sample size OR if no sample-size calculation was performed, describe how sample sizes were chosen and provide a rationale for why these sample sizes are sufficient.*
Data collection	*Describe the data collection procedure, including who recorded the data and how.*
Timing and spatial scale	*Indicate the start and stop dates of data collection, noting the frequency and periodicity of sampling and providing a rationale for these choices. If there is a gap between collection periods, state the dates for each sample cohort. Specify the spatial scale from which the data are taken*
Data exclusions	*If no data were excluded from the analyses, state so OR if data were excluded, describe the exclusions and the rationale behind them, indicating whether exclusion criteria were pre-established.*
Reproducibility	*Describe the measures taken to verify the reproducibility of experimental findings. For each experiment, note whether any attempts to repeat the experiment failed OR state that all attempts to repeat the experiment were successful.*
Randomization	*Describe how samples/organisms/participants were allocated into groups. If allocation was not random, describe how covariates were controlled. If this is not relevant to your study, explain why.*
Blinding	*Describe the extent of blinding used during data acquisition and analysis. If blinding was not possible, describe why OR explain why blinding was not relevant to your study.*

Did the study involve field work? ☐ Yes ☐ No

Field work, collection and transport

Field conditions	*Describe the study conditions for field work, providing relevant parameters (e.g. temperature, rainfall).*
Location	*State the location of the sampling or experiment, providing relevant parameters (e.g. latitude and longitude, elevation, water depth).*
Access and import/export	*Describe the efforts you have made to access habitats and to collect and import/export your samples in a responsible manner and in compliance with local, national and international laws, noting any permits that were obtained (give the name of the issuing authority, the date of issue, and any identifying information).*
Disturbance	*Describe any disturbance caused by the study and how it was minimized.*

Reporting for specific materials, systems and methods

We require information from authors about some types of materials, experimental systems and methods used in many studies. Here, indicate whether each material, system or method listed is relevant to your study. If you are not sure if a list item applies to your research, read the appropriate section before selecting a response.

Materials & experimental systems

n/a	Involved in the study	
☐	☒	Antibodies
☐	☒	Eukaryotic cell lines
☒	☐	Palaeontology
☐	☒	Animals and other organisms
☒	☐	Human research participants
☒	☐	Clinical data

Methods

n/a	Involved in the study	
☒	☐	ChIP-seq
☐	☒	Flow cytometry
☒	☐	MRI-based neuroimaging

Antibodies

Antibodies used

Antibodies against the following proteins were used for Western blotting: beta-ACTIN (1:5,000, Sigma Aldrich, A2228), HIF-1a (1:500, Novus Biologicals, NB-100-479), LAMIN-A/C (1:500, Santa Cruz, sc-376248), LDH-A (1:500, Santa Cruz Biotechnology, sc-27230), PDK1 (1:1,000, Assay Designs, KAP-PK112-0), VHL (1:1,000, Cell Signaling Technologies, #68547), VINCULIN (1:5,000, Abcam, ab130007).

For immunohistochemistry, primary antibodies against the following proteins or epitopes were used at the following dilutions and antigen retrieval conditions: B220 (1:3,000, BD Biosciences, 553084, Tris/EDTA 20 min, 100oC), CA9 (1:2,000, Invitrogen, PA1-16592, citrate, 10 min, 110oC), CD3 (1:250, Zytomed, RBK024, citrate 30 min, 95oC), CD4 (1:1,000, eBioscience, 14-9766, citrate, 30 min, 100oC), CD8a (1:200, Invitrogen, 14-0808-82, citrate buffer, 15 min, 114oC), CD10 (1:2,000, Thermo Fisher Scientific, PA5-47075, citrate buffer, 10 min, 110oC), CD68 (1:100, abcam ab125212, citrat, 30min), CD69 (1:1,000, Bioss, bs-2499R, Tris/EDTA, 15 min, 114 oC), F4/80 (1:250, Linaris Biologische Produkte, T-2006, BOND Enzyme Pretreatment Kit (Leica AR9551), 10 min, 37 oC), HIF-1a (1:20,000, Novus Biotechnologies, NB-100-105, citrate buffer, 10 min, 110oC, Catalyzed Signal Amplification Kit (DakoCytomation)), HIF-2a (1:1,000, abcam ab109616, Tris/EDTA 15 min, 114 oC), Ly-6G (1:800, BD, 551459), MHC II (1:500, Novus Biotechnologies, NBP1-43312, BOND Enzyme Pretreatment Kit (Leica AR9551), 10 min, 37 oC), PD-1 (1:100, R&D systems, AF1021, Tris/EDTA 20 min, 100oC), Perforin (1:100, Biorbyt, orb312827, Tris/EDTA, 15 min, 114 oC), phospho-Thr37/Thr46-4E-BP1 (1:800, Cell Signaling Technologies, 2855, citrate buffer, 10 min, 110oC).

Antibodies used in T cell proliferation assay:

CD16/32 antibody (Fisher Scientific, 14016185, diluted 1:25 in MACS buffer)

CD8a antibody (APC-conjugated, Biolegend, 100712, diluted 1:100 in MACS buffer)

Validation

Antibodies used for Western-blotting (see above):

1. beta-ACTIN (Sigma Aldrich, A2228, Validation: Well characterised in the literature e.g. Gimona, M., et al., Cell Motil. Cytoskel., 27, 108-116 (1994).)
2. HIF-1a(Novus Biologicals, NB-100-479, Validation via Biological Strategies by manufacturer: These strategies use defined biological or chemical modulation of protein expression to demonstrate antibody specificity to the target protein. The data is compared across multiple cell lines including positive and negative expressing cells, and multiple species, if applicable.)
3. LAMIN-A/C (Santa Cruz, sc-376248, Validation: Well characterised in the literature e.g. Ruchaud, S., et al., EMBO J., 21, 1967-1977(2002))
4. LDH-A (Santa Cruz Biotechnology, sc-27230, Validation: Well characterised in the literature e.g. Esen, E. et al. 2013. Cell Metab. 17: 745-755)
5. PDK1 (Assay Designs, KAP-PK112-0, Validation: Well characterised in the literature e.g. T. Golias, et al.; Sci. Rep. 6, 31146 (2016))
6. VHL (Cell Signaling Technologies, #68547, Validation: specificity is shown in our study using genetic knockout)
7. VINCULIN (Abcam, ab130007, Validation: Well characterised in the literature e.g. Demircioglu FE et al. Nat Commun 10:3262 (2019))

Antibodies used for immunohistochemistry stainings (see above):
1. B220 (BD Biosciences, 553084, Validation: In house validation was performed using lymphoid tissue)
2. CA9 (Invitrogen, PA1-16592, Validation: Cell Treatment Antibody Validation was performed by company, specificity was further confirmed by our VpRH1-knockout genotype)
3. CD3 (Zytomed, RBK024, Validation: In house validation was performed using lymphoid tissue)
4. CD4 (eBioscience, 14-9766, Validation: In house validation was performed using lymphoid tissue)
5. CD8a (Invitrogen, 14-0808-82, Validation: In house validation was performed using lymphoid tissue)
6. CD10 (Thermo Fisher Scientific, PA5-47075, Validation: Relative Expression Antibody Validation was performed by company)
7. CD68 (abcam ab125212, Validation: Well characterised in the literature e.g. Zheng L et al. J Cell Mol Med 24:1276-1285 (2020), In house validation was performed using lymphoid tissue)
8. CD69 (Bioss, bs-2499R, Validation: Characterised in the literature e.g. Mokuda et al. (2015) Arthritis.Res.Ther. 17:275)
9. F4/80 (Linaris Biologische Produkte, T-2006, Validation: In house validation was performed using lymphoid tissue)
10. HIF-1a (Novus Biotechnologies, NB-100-105, Validation by company via biological and genetic strategies, In house specificity validation using VpRH1-knockout genotype)
11. HIF-2a (abcam ab109616, Validation: In house validation with genetic HIF2a-KO)
12. Ly-6G (1:800, BD, 551459, Validation: In house validation was performed using lymphoid tissue)
13. MHC II (Novus Biotechnologies, NBP1-43312, Validation: In house validation was performed using lymphoid tissue)
14. PD-1 (R&D systems, AF1021, Validation: Well characterizesed in the literature i.e. Coyle, A. and J. Gutierrez-Ramos (2001) Nat. Immunol. 2:203.)
15. Perforin (Biorbyt, orb312827, Validation: In house validation was performed using lymphoid tissue)
16. phospho-Thr37/Thr46-4E-BP1 (Cell Signaling Technologies, 2855, Validation: Well characterizesed in the literature i.e. Rambur, A., Lours-Calet, C., et al. Nature Communications 2020)

Antibodies used in T cell proliferation assay:
1. CD16/32 antibody (Fisher Scientific, 14016185, Validation: Well characterizesed in the literature i.e. Lizotte PH et. al. Oncoimmunology 2019)
2. CD8a antibody (Biolegend, 100712, Well characterizesed in the literature i.e. Validation: Zamoyska R. 1994. Immunity 1:243.)

Eukaryotic cell lines

Policy information about cell lines

Cell line source(s)	Primary MEFs and the mouse ccRCC cell lines used in this study were generated in our laboratory. Human RPTECs were obtained from Dr Jiin-Kuan Yee, 786-O and A498 cells were from ATCC.
Authentication	Western blotting and/or real time PCR were used to validate the genotype of cells generated in our laboratory. RPTEC cells were confirmed by STR PCR analyses at 21 loci and by the presence of T-antigen by western blotting. 786-O and A498 cells were validated by the supplier and western blotting for VHL and SETD2 confirmed the predicted patterns of protein expression based on the distinctive gene mutations present in these cell lines.
Mycoplasma contamination	All cells used in this study tested negative for contamination by mycoplasma.
Commonly misidentified lines (See ICLAC register)	No commonly misidentified cell lines were used in the study.

Palaeontology

Specimen provenance	*Provide provenance information for specimens and describe permits that were obtained for the work (including the name of the issuing authority, the date of issue, and any identifying information).*
Specimen deposition	*Indicate where the specimens have been deposited to permit free access by other researchers.*
Dating methods	*If new dates are provided, describe how they were obtained (e.g. collection, storage, sample pretreatment and measurement), where they were obtained (i.e. lab name), the calibration program and the protocol for quality assurance OR state that no new dates are provided.*

☐ Tick this box to confirm that the raw and calibrated dates are available in the paper or in Supplementary Information.

Animals and other organisms

Policy information about studies involving animals; ARRIVE guidelines recommended for reporting animal research

Laboratory animals	Ksp1.3-CreERT2;Vhlfl/fl;Trp53fl/fl;Rb1fl/fl mice were intercrossed with Ksp1.3-CreERT2;Vhlfl/fl;Trp53fl/fl;Hif1afl/fl and Ksp1.3-CreERT2;Vhlfl/fl;Trp53fl/fl;Hif2afl/fl mice to generate the experimental Ksp1.3-CreERT2;Vhlfl/fl;Trp53fl/fl;Rb1fl/fl, Ksp1.3-CreERT2;Vhlfl/fl;Trp53fl/fl;Rb1fl/flHif1afl/fl and Ksp1.3-CreERT2;Vhlfl/fl;Trp53fl/fl;Rb1fl/flHif2afl/fl mouse lines. Littermate mice that lacked the Cre transgene served as wild type controls. Gene deletion in 6 week-old mice was achieved by feeding with food containing tamoxifen (400 parts per million) for 2 weeks. Mouse crosses and phenotyping were conducted under the breeding license of the Laboratory Animal Services Center, University of Zurich and tumour monitoring studies were conducted under license ZH116/16 of the Canton of Zurich. The environmental conditions in the mouse facility were: 12h light and 12h dark cycle,

	Temperature range of 21-23°C and humidity range of 40-50%.
Wild animals	The study did not involve wild animals.
Field-collected samples	The study did not involve samples from the field.
Ethics oversight	Mouse crosses and phenotyping were conducted under the breeding license of the Laboratory Animal Services Center, University of Zurich and tumour monitoring studies were conducted under license ZH116/16 of the Canton of Zurich. Mouse allograft experiments were conducted under license G-17/165 of the Regierungspräsidium Freiburg.

Note that full information on the approval of the study protocol must also be provided in the manuscript.

Human research participants

Policy information about studies involving human research participants

Population characteristics	*Describe the covariate-relevant population characteristics of the human research participants (e.g. age, gender, genotypic information, past and current diagnosis and treatment categories). If you filled out the behavioural & social sciences study design questions and have nothing to add here, write "See above."*
Recruitment	*Describe how participants were recruited. Outline any potential self-selection bias or other biases that may be present and how these are likely to impact results.*
Ethics oversight	*Identify the organization(s) that approved the study protocol.*

Note that full information on the approval of the study protocol must also be provided in the manuscript.

Clinical data

Policy information about clinical studies

All manuscripts should comply with the ICMJEguidelines for publication of clinical research and a completedCONSORT checklist must be included with all submissions.

Clinical trial registration	*Provide the trial registration number from ClinicalTrials.gov or an equivalent agency.*
Study protocol	*Note where the full trial protocol can be accessed OR if not available, explain why.*
Data collection	*Describe the settings and locales of data collection, noting the time periods of recruitment and data collection.*
Outcomes	*Describe how you pre-defined primary and secondary outcome measures and how you assessed these measures.*

ChIP-seq

Data deposition

- [ ] Confirm that both raw and final processed data have been deposited in a public database such as GEO.
- [ ] Confirm that you have deposited or provided access to graph files (e.g. BED files) for the called peaks.

Data access links<br>*May remain private before publication.*	*For "Initial submission" or "Revised version" documents, provide reviewer access links. For your "Final submission" document, provide a link to the deposited data.*
Files in database submission	*Provide a list of all files available in the database submission.*
Genome browser session<br>(e.g. UCSC)	*Provide a link to an anonymized genome browser session for "Initial submission" and "Revised version" documents only, to enable peer review. Write "no longer applicable" for "Final submission" documents.*

Methodology

Replicates	*Describe the experimental replicates, specifying number, type and replicate agreement.*
Sequencing depth	*Describe the sequencing depth for each experiment, providing the total number of reads, uniquely mapped reads, length of reads and whether they were paired- or single-end.*
Antibodies	*Describe the antibodies used for the ChIP-seq experiments; as applicable, provide supplier name, catalog number, clone name, and lot number.*
Peak calling parameters	*Specify the command line program and parameters used for read mapping and peak calling, including the ChIP, control and index files used.*
Data quality	*Describe the methods used to ensure data quality in full detail, including how many peaks are at FDR 5% and above 5-fold enrichment.*

Software

Describe the software used to collect and analyze the ChIP-seq data. For custom code that has been deposited into a community repository, provide accession details.

Flow Cytometry

Plots

Confirm that:

- [x] The axis labels state the marker and fluorochrome used (e.g. CD4-FITC).
- [x] The axis scales are clearly visible. Include numbers along axes only for bottom left plot of group (a 'group' is an analysis of identical markers).
- [x] All plots are contour plots with outliers or pseudocolor plots.
- [x] A numerical value for number of cells or percentage (with statistics) is provided.

Methodology

Sample preparation

10,000 mouse ccRCC 2020, human RPTEC, 786-0 or A498 ccRCC cells were seeded in triplicates in a 6-well-plate with 2 ml RPMI + 10% FCS and kept incubated in a humidified 5% (v/v) CO2 and 20% O2 incubator at 37°C for two days. Two days later, spleens from C57BL/6 mice were extracted, washed in PBS and mashed through a 100 µm cell strainer in MACS buffer (PBS 1x + 2 % FCS + 2 mM EDTA). The mashed spleen was filtered again through a 100 µm cell strainer into a 50 ml conical tube and centrifugated for 10 minutes at 1200 rpm. The pellet was labelled manually with magnetic CD8a (Ly-2) MicroBeads (Miltenyi Biotech). Isolated CD8a+ T cells were centrifuged, resuspended in proliferation medium (RPMI + 10% FCS supplemented with 25 µM-Mercaptoethanol) and counted. CD8a+ T cells were then stained with the CellTrace Violet Proliferation Dye (Thermo Fisher). Stained CD8a+ T cells were stimulated with CD3/CD28 Dynabeads (Thermo Fisher) and activated with interleukin-2 (IL-2). The conditioned medium was distributed into fresh 6-well-plates and 2x105 of stained, stimulated and activated CD8a+ T cells were added. The mix of conditioned medium and T-cells was incubated for three days in a humidified 5% (v/v) CO2 and 20% O2 incubator at 37°C. After the incubation time the T cells were resuspended and centrifuged in a 2 ml reaction tube for 5 minutes at 1600 rpm and 4 °C. The dead cells within the pellet were stained with the Live/Dead Fixable Aqua Dead Cell Stain Kit (Thermo Fisher), washed with 200 µl MACS buffer and centrifuged for 5 minutes at 1600 rpm and 4 °C, 25 µl CD16/32 antibody (Fisher Scientific, 14016185, diluted 1:25 in MACS buffer) was added to the pellet to block Fc-mediated reactions. After 10 minutes of incubation at 4 °C in the dark, 25 µl of CD8a antibody (APC-conjugated, Biolegend, 100712, diluted 1:100 in MACS buffer) was added to the suspension and incubated for 30 minutes at 4 °C in the dark. Afterwards T cells were washed twice with MACS buffer and the pellet was resuspended in 100 µl MACS buffer.

Instrument

BD LSRFortessa Cell Analyzer Cat. No. 649225

Software

FlowJo V10.4

Cell population abundance

Numbers and post-sort fractions (% alive cells, absolute number, % CD8+ cells, absolute number):
Supplementary Figure 12c
1. Stained, not activated (45, 23287, 78.8, 18347)
2. Unstained, not activated (58.8, 2106, 67.5, 1421)
3. Non-conditioned medium (7.8, 2840, 67.6, 1919)
4. 2020 shRNA-Hif1a #220 (11.4, 3673, 84.3, 3097)
5. 2020 shRNA-Hif1a #222 (11.7, 4960, 80.1, 3974)
6. 2020 shRNA-ns (8.1, 2537, 74.6, 1892)
7. 2020 VHL30 (10.1, 3528, 66.5, 2345)
8. 2020 Vector (6.63, 2419, 68.3, 1653)

Supplementary Figure 12c
1. 786-O + VHL30 (20.5, 9040, 88.9, 8036)
2. 786-O (20, 8740, 91, 7956)
3. RPTEC (21.6, 9135, 89.1, 8143)
4. A498 (16.8, 6782, 85.5, 5801)
5. 2020 (24.4, 8759, 82.6, 7231)
6. Non-conditioned medium (10.5, 3825, 80.2, 3066)
7. Stained, not activated (45, 23287, 78.8, 18347)

Gating strategy

1. Dead/living cells were measured with a 405 nm Extinction Laser (AmCyan)
2. T cells were measured with a 640 nm Extinction Laser (APC)
3. Proliferation Dye was measured with a 405 nm Extinction Laser (Pacific Blue).

- [x] Tick this box to confirm that a figure exemplifying the gating strategy is provided in the Supplementary Information.

Magnetic resonance imaging

Experimental design

Design type	*Indicate task or resting state; event-related or block design.*
Design specifications	*Specify the number of blocks, trials or experimental units per session and/or subject, and specify the length of each trial or block (if trials are blocked) and interval between trials.*
Behavioral performance measures	*State number and/or type of variables recorded (e.g. correct button press, response time) and what statistics were used to establish that the subjects were performing the task as expected (e.g. mean, range, and/or standard deviation across subjects).*

Acquisition

Imaging type(s)	*Specify: functional, structural, diffusion, perfusion.*
Field strength	*Specify in Tesla*
Sequence & imaging parameters	*Specify the pulse sequence type (gradient echo, spin echo, etc.), imaging type (EPI, spiral, etc.), field of view, matrix size, slice thickness, orientation and TE/TR/flip angle.*
Area of acquisition	*State whether a whole brain scan was used OR define the area of acquisition, describing how the region was determined.*

Diffusion MRI ☐ Used ☐ Not used

Preprocessing

Preprocessing software	*Provide detail on software version and revision number and on specific parameters (model/functions, brain extraction, segmentation, smoothing kernel size, etc.).*
Normalization	*If data were normalized/standardized, describe the approach(es): specify linear or non-linear and define image types used for transformation OR indicate that data were not normalized and explain rationale for lack of normalization.*
Normalization template	*Describe the template used for normalization/transformation, specifying subject space or group standardized space (e.g. original Talairach, MNI305, ICBM152) OR indicate that the data were not normalized.*
Noise and artifact removal	*Describe your procedure(s) for artifact and structured noise removal, specifying motion parameters, tissue signals and physiological signals (heart rate, respiration).*
Volume censoring	*Define your software and/or method and criteria for volume censoring, and state the extent of such censoring.*

Statistical modeling & inference

Model type and settings	*Specify type (mass univariate, multivariate, RSA, predictive, etc.) and describe essential details of the model at the first and second levels (e.g. fixed, random or mixed effects; drift or auto-correlation).*
Effect(s) tested	*Define precise effect in terms of the task or stimulus conditions instead of psychological concepts and indicate whether ANOVA or factorial designs were used.*

Specify type of analysis: ☐ Whole brain ☐ ROI-based ☐ Both

Statistic type for inference (See Eklund et al. 2016)	*Specify voxel-wise or cluster-wise and report all relevant parameters for cluster-wise methods.*
Correction	*Describe the type of correction and how it is obtained for multiple comparisons (e.g. FWE, FDR, permutation or Monte Carlo).*

Models & analysis

n/a	Involved in the study
☐	☐ Functional and/or effective connectivity
☐	☐ Graph analysis
☐	☐ Multivariate modeling or predictive analysis

Functional and/or effective connectivity	*Report the measures of dependence used and the model details (e.g. Pearson correlation, partial correlation, mutual information).*
Graph analysis	*Report the dependent variable and connectivity measure, specifying weighted graph or binarized graph, subject- or group-level, and the global and/or node summaries used (e.g. clustering coefficient, efficiency, etc.).*

Multivariate modeling and predictive analysis

Specify independent variables, features extraction and dimension reduction, model, training and evaluation metrics.
